# Alternative polyadenylation associated with prognosis and therapy in colorectal cancer

**DOI:** 10.1038/s41598-022-11089-9

**Published:** 2022-04-29

**Authors:** Yi Zhang, Yunfei Xu, Yuzhi Wang

**Affiliations:** 1 Department of Blood Transfusion, People’s Hospital of Deyang City, No. 173 North Taishan Road, Deyang, 618000 Sichuan China; 2 Department of Laboratory Medicine, Chengdu Women’s and Children’s Central Hospital, No. 1617 Sun Moon Avenue, Chengdu, 610031 Sichuan China; 3 Department of Laboratory Medicine, People’s Hospital of Deyang City, No. 173 North Taishan Road, Deyang, 618000 Sichuan China

**Keywords:** Cancer, Computational biology and bioinformatics, Molecular biology, Biomarkers, Gastroenterology, Molecular medicine, Oncology, Risk factors

## Abstract

Colorectal cancer (CRC) is among the most widely spread cancers globally. Aberrant alternative polyadenylation (APA) plays a role in cancer onset and its progression. Consequently, this study focused on highlighting the role of APA events and signals in the prognosis of patients with CRC. The APA events, RNA sequencing (RNA-seq), somatic mutations, copy number variants (CNVs), and clinical information of the CRC cohort were obtained from The Cancer Genome Atlas (TCGA) database and UCSC (University of California-Santa Cruz) Xena database. The whole set was sorted into two sets: a training set and a test set in a ratio of 7:3. 197 prognosis-related APA events were collected by performing univariate Cox regression signature in patients with CRC. Subsequently, a signature for APA events was established by least absolute shrinkage and selection operator (LASSO) and multivariate Cox analysis. The risk scores were measured for individual patients on the basis of the signature and patients were sorted into two groups; the high-risk group and the low-risk group as per their median risk scores. Kaplan–Meier curves, principal component analysis (PCA), and time-dependent receiver operator characteristic (ROC) curves revealed that the signature was able to predict patient prognosis effectively and further validation was provided in the test set and the whole set. The high-risk and low-risk groups displayed various distributions of mutations and CNVs. Tumor mutation burden (TMB) alone and in combination with the signature predicted the prognosis of CRC patients, but the gene frequencies of TMBs and CNVs did not change in the low- and high-risk groups. Moreover, immunotherapy and chemotherapy treatments showed different responses to PD-1 inhibitors and multiple chemotherapeutic agents in the low and high-risk groups based on the tumor immune dysfunction and exclusion (TIDE) and genomics of drugs sensitivity in cancer (GDSC) databases. This study may help in understanding the potential roles of APA in CRC, and the signature for prognosis-related APA events can work as a potential predictor for survival and treatment in patients with CRC.

## Introduction

Colorectal cancer (CRC) is listed among the most widely known malignancies around the globe. The latest epidemiological statistical analysis indicated that there were roughly 1.9 million new cases of CRC and 935,000 CRC-related mortalities around the world in 2015^1^. At present, the major treatment methods for CRC comprise laparoscopic surgery, radiotherapy, and palliative chemotherapy^2^. Since CRC is known to be asymptomatic most of the time and hard to detect in its initial stages, most patients reach an advanced stage by the time they are diagnosed^3^. Patients with advanced stage of CRC show a much poorer response to treatment and overall survival (OS) as compared to those who are in the early stages of CRC. The 5-year OS for patients with advanced CRC is 10%^4^. In a clinical setting, the carcinoembryonic antigen (CEA) and carbohydrate antigen 199 (CA199) are commonly used as CRC markers, but they show less sensitivity and reduced specificity for the diagnosis and prognostic assessment of CRC, particularly in patients with early-stage CRC^5^. Thus, understanding the underlying molecular mechanisms in the occurrence and progression of CRC and identifying new biomarkers is significant for the diagnosis, treatment, and prognosis of patients with CRC.

Alternative polyadenylation (APA) is a substantial mechanism for transcript and protein level regulation. About 70 percent of the genes in humans contain multiple polyadenylation [poly(A)] loci, typically in the 3′ untranslated regions (3′ UTR) of the Messenger RNAs (mRNAs), leading to the formation of transcript isomers having variable lengths and contents^6,7^. The choice of APA loci can be determined by extracellular signals and the protein components available for involvement in the mechanism of APA. The mRNA transcriptional sequences created by APA have a role in the regulation of events like development, differentiation, and other physiological conditions^8^. According to recent research, the dysregulation of APA causes a variety of human diseases, including cancer^8^. Included in the APA events that are known to be dysregulated in different types of tumors, 3′ UTR-shortened events (61–98% of total APA events) are important in this regard^9,10^. These 3′ UTR-shortened events are involved in the regulation of the expression of cancer-related genes through the loss of regulatory loci of the microRNA (miRNA)^11,12^. Moreover, shortened APA events regulate oncogene expression by lowering the competitive endogenous RNA interference^13,14^. Past research has shown that there is a close association between APA events and CRC^15^, but a systematic analysis of the impact of APA events regarding the prognosis and treatment of patients with CRC is not sufficient.

The representative technologies for detecting APA are polyadenylation signal site sequence, (PAS-seq), Passeq and 3 'Extraction and Depth Sequence (3′READS)^7,16^. However, they have disadvantages such as high cost and complex analysis, making it difficult to obtain APA data with large samples. Recently, the transcriptome analysis algorithm based on RNA-seq data analysis APA has made great progress. The dynamic analysis of alternative polyadenylation from RNA-Seq (DaPars) is the most widely used bioinformatics algorithm for analyzing APA based on deep RNA-seq data^9^. The DaPars algorithm identified the distal PAS and predicted proximal PAS localization based on a specific linear regression model. The algorithm applies the percentage of distal polyA site usage Index (PDUI) values to quantitative changes of APA, ranging from 0 to 1. The larger the PDUI value, the more polyadenylation sites at the distal end of transcript. This makes it possible to quantify and excavate APA with large scale samples based on the depth sequencing data (including public databases), as well as explore their potential clinical and biological significance. This study aimed at identifying APA events in CRC and highlighting their prognostic significance in patients with CRC using data obtained from The Cancer Genome Atlas (TCGA) database. Subsequently, this study identified APA events that were strongly associated with CRC prognosis and generated the APA-related prognostic signature. Furthermore, we assessed the correlation between the signature and somatic mutations, CNVs, tumor immune microenvironment (TIM), and immunotherapy. Additionally, a variety of potential drugs against this signature were screened using publicly available drug sensitivity databases. In conclusion, a core regulators-alternative splicing (CRs-APA) regulatory network was constructed, showing a potential association between CRs and prognosis-related APA in CRC.

## Materials and methods

### Data acquisition and pre-processing

Gene expression profiles, somatic mutations, data on CNVs, and corresponding clinical information (Supplementary Table 1) for CRC patients were provided by the TCGA data portal website. The corresponding APA data of the same TCGA cohort were downloaded from the UCSC Xena database. To generate a reliable APA dataset, this study screened APA events under the following criteria: (I) Percentage of samples with PDUI value ≥ 75% (II) mean of PDUI values ≥ 0.05; (III) standard deviation of PDUI values ≥ 0.05. Missing PDUI values were complemented using the k nearest neighbors’ algorithm^17^. Patients with OS lower than 28 days were eliminated to reduce the impact of patients who died due to non-tumor factors. Furthermore, we randomized whole set (n = 383) in a 7:3 ratio to the training set (n = 270) and the test set (n = 113). Randomization was computer generated centrally by a single sequence of random assignments to decrease subgroup bias as much as possible. The data utilized in our study were obtained from public databases, so ethical approval was not necessary. Besides, all methods were carried out in accordance with relevant guidelines and regulations.

### Identification of survival-related APA events, functional enrichment analysis, and gene network construction

For the assessment of the connection between APA events and OS, a univariate Cox regression analysis was carried out on the training set for the purpose of identifying the survival-related APA events. Additionally, possible mechanisms underlying the occurrence of APA events in CRC were explored by performing the Gene Ontology (GO) and Kyoto Encyclopedia of Genes and Genomes (KEGG) pathway enrichment analyses analysis using Metascape web tool (https://metascape.org/gp/index.html)^18^ on the parental genes of survival-related APA events, such as biological process, cellular component, and molecular function. A false discovery rate (FDR) < 0.05 was set as significant. A previous report has identified 22 genes as CRs of APA^19^. To identify their regulatory roles in APA events, the correlation between CRs and prognostic-related APA events was assessed using Pearson correlation analysis, and a regulatory network map was built with the aid of the Cytoscape software (http://www.cytoscape.org)^20^. The |correlation coefficients| > 0.4 and P < 0.001 of the PDUI values of APA events and the expression of CRs were used as screening thresholds for relationship pairs.

### Construction and assessment of prognostic signature

Initially, a least absolute shrinkage and selection operator (LASSO) regression was conducted on the top 20 most prognosis-related APA events to exclude false-positive parameters that can occur due to overfitting. Subsequently, these APA events were used to create the prognostic prediction signature using multivariate regression analysis. The following formula was used for creating a prognostic score for these APA events: Risk score = $\sum\nolimits_{k = 1}^{n} {}$ (PDUI_k_ × coef_k_). n denotes the total number of considered APA events, i = 1 denotes that the formula is incorporated from the first APA event. Where PDUI_k_ is the PDUI value of APA events for patient k and the coef_k_ is the regression coefficient of APA events for patient k. The patients included in the training set were then scored and sorted into the high and low-risk groups based on their median risk scores. Kaplan–Meier (KM) analysis was performed to analyze the survival probability in both these groups and Log-rank tests were carried out for the purpose of comparing the survival differences. 1, 3, and 5-year time-dependent receiver operator characteristic (ROC) curves were plotted to verify the predictive effectiveness of the signature, and then the area under the curve (AUC) values were measured. Moreover, principal component analysis (PCA) was used for testing the variations of patients in both groups.

### Analysis of mutations and CNVs

Tumor mutation burden (TMB) is the number of somatic base substitution and indels with a potential effect on protein function (nonsynonymous and frameshifts) per megabase, considering only variants with a frequency > 0.05. The “maftools” (https://www.bioconductor.org/packages/release/bioc/vignettes/maftools) in the R package helped in visualizing the mutation landscape of the whole set and to measuring the TMB for individual patients^21^. The gain and loss levels regarding CNVs were identified using segmentation analysis and the online web tool “GISTIC 2.0” (https://cloud.genepattern.org, version 6.15.28)^22^, followed by a difference comparison in CNVs between the high and low-risk groups.

### Exploration of immune microenvironment

Initially, the immune scores, stromal scores, ESTIMATE scores, and tumor purity assessment was performed with the help of the 'ESTIMATE' package (https://bioinformatics.mdanderson.org/estimate)^23^ and then they were all compared between the high and low-risk groups. The expression characteristics of 24 immune cells from earlier studies were then analyzed with the help of the 'GSVA' package (http://www.bioconductor.org/packages/release/bioc/html/GSVA)^24^ and single-sample gene set enrichment analysis (ssGSEA)^25^. The enrichment scores for each of the 24 immune cells signatures represent the absolute enrichment of a specific set of genes in each sample in the dataset^26^. Finally, the expression levels of 14 potentially targeted immune checkpoint molecules were compared between the low- and high-risk groups^27^.

### Estimation of immunotherapy and chemotherapy

As accessible open data is not available on CRC cohorts undergoing both APA testing and immunotherapy, initially the response of the TCGA-CRC cohort to immunotherapy was determined by the TIDE algorithm^28^. The targeted drugs under the signature were screened using a ridge regression signature according to the GDSC cell line expression profile (http://www.cancerrxgene.org) and the gene expression profiles of CRC patients was constructed using the 'pRRophetic' in R package for the purpose of predicting the half-maximal inhibitory concentration (IC50) values of the compound/inhibitor.

### Estimation of immunotherapy and chemotherapy

For the identification of factors with independent prognostic value, univariate and multivariate Cox regression analyses were carried out on these clinical manifestations and risk scores. Afterward, nomogram construction was completed using the Risk Management Signature (RMS) (http://www.rms.com) in the R package for visualization and the prediction of 1, 3, and 5-year survival rates according to the independent prognostic factors. The calibrated chart, concordance index (C-index) curves, and time-AUC curves were used to assess the differentiating power and accuracy of the nomogram. In the end, prediction of the clinical outcome variables and quantification of the clinical utility of the nomogram was conducted with the help of decision curve analysis (DCA) curves.

### Exploration and validation of the APAs in an independent dataset

In order to validate the 3′UTR difference in APAs of the signature between normal and cancer tissues. Another dataset (GSE50760), which contains the RNA-seq raw data (SRA files) from CRC (n = 36) and normal controls tissues (n = 18), was downloaded from the GEO database (https://www.ncbi.nlm.nih.gov/geo/). DaPars algorithm was used to identify and quantified APA events for each sample. The differential APA events were defined by the Benjamini–Hochberg adjusted P-value < 0.05. Meanwhile, the immune status and therapy response for all patients within this cohort were evaluated by the same above approaches (ESTIMATE algorithm, ssGSEA algorithm, TIDE algorithm and GDSC database).

### Statistical analysis

The statistical analyses in the present research were done with the help of R (version: 4.0.1). The Wilcoxon test was performed for the comparison between the two groups of patients, and the Kruskal–Wallis test was done for comparing data of multiple groups. Correlation analysis between the high-risk group and the low-risk group and the analysis of clinicopathological information was performed by a chi-square test and Spearman analysis was carried out to determine their correlation coefficients. The two-tailed P < 0.05 was set as a significant value if not stated otherwise.

## Results

### Identification and correlation analysis of prognosis-related APA events

**Figure 1:**
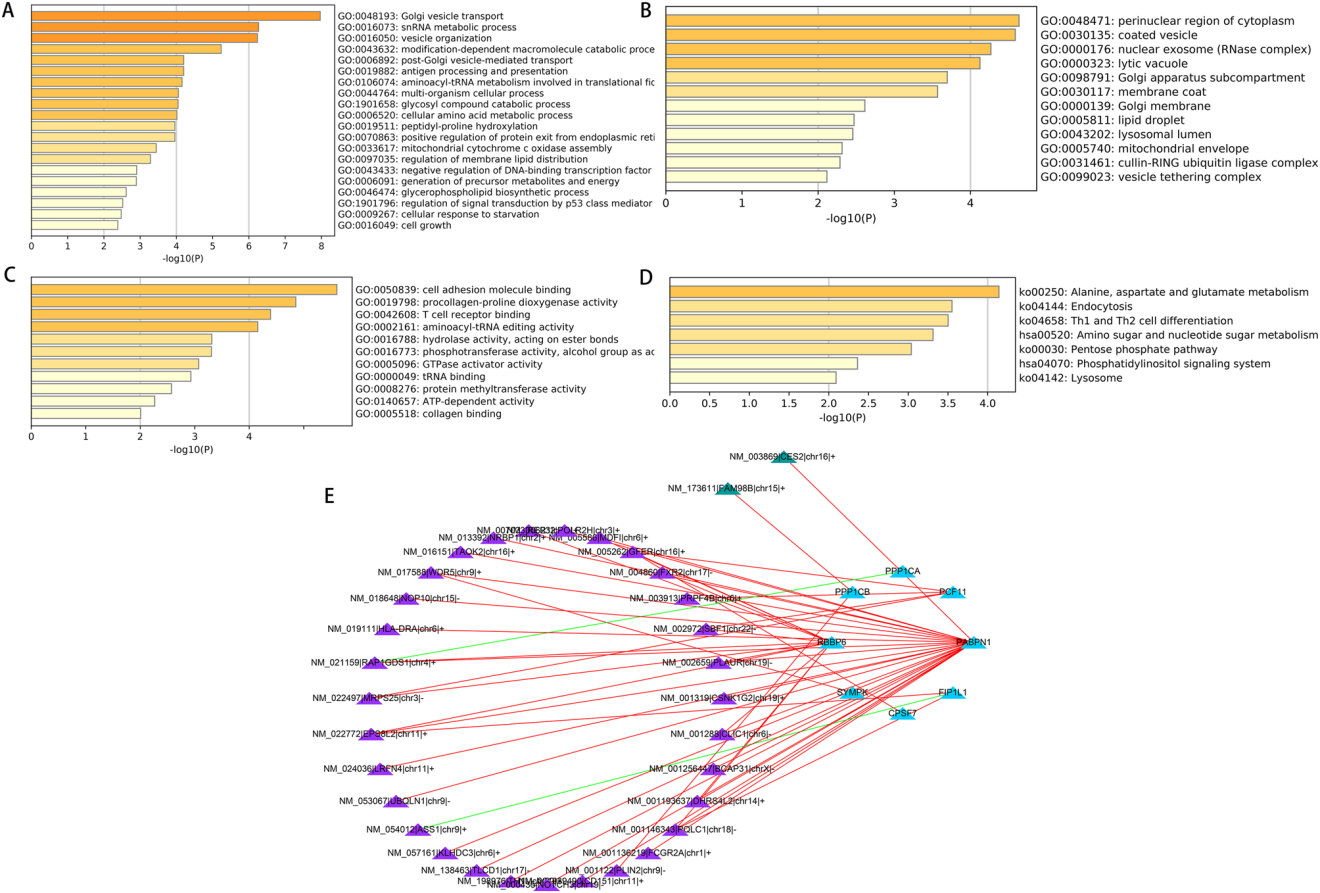
Enrichment analysis of the corresponding genes of prognosis-related APA events and Construction of a survival-associated. Volcano plot of prognosis-related APA events. An overview of the GO annotations of the prognostic APA in three categories: BP (**A**), CC (**B**) and MF (**C**). (**D**) KEGG pathway analysis. (**E**) CRs-APAs network (blue triangles, purple triangles and green triangles were CRs, poor prognosis events and good prognosis events, respectively. Red/green lines represent positive/negative correlations between nodes).

383 CRC patients were included in this study. Moreover, 5530 APA events were identified from 5294 genes based on the screening criteria mentioned above (Supplementary Table 2). All patients included in this study were randomly sorted into two sets called the training set and the test set in a 7:3 ratio. The clinical and pathological manifestations of all the patients are summarized in Table 1. For prognostic value identification value of APA events in patients with CRC, the assessment of the prognostic effect of APA events was performed with the help of univariate Cox regression analysis. Overall, 197 APA events were explored to be substantially correlated with OS (Fig. S1, Supplementary Table 3). Subsequently, biological functional enrichment analysis was carried out for the assessment of the potential impact of these events on the parental genes. Figure 1A–C illustrates that the parental genes of these events were enriched in specific GO categories, including cell adhesion molecule binding, perinuclear region of cytoplasm, and Golgi vesicle transport. Additionally, prognosis-related APA events enriched several pathways for these genes such as the Pentose phosphate pathway and Endocytosis (Fig. 1D). The outcomes of the enrichment analysis indicated a close correlation among prognostic-related parental genes in APA events and the progression of CRC. In the correlation network constructed with the help of Cytoscape (http://www.cytoscape.org), the correlation of CRs genes and APA events was not just one-to-one, but many-to-one or one-to-many (Fig. 1E, Supplementary Table 4). Furthermore, CRs exerted only a positive correlation with the low-risk events, while they showed both positive and negative correlation effects on high-risk events.

**Table 1:** Clinical characteristics of the colorectal cancer patients.

Characteristics	Groups	Number (percentage)
Age	< 60	109 (28%)
	> 60	274 (72%)
Gender	Male	206 (54%)
	Female	177 (46%)
T	T1–T2	77 (20%)
	T3–T4	305 (80%)
N	N0	228 (60%)
	N1–N3	155 (40%)
M	M0	281 (84%)
	M1	53 (16%)
Stage	Stage I–II	214 (57%)
	Stage III–IV	159 (43%)
Lymph node count	< 20	184 (51%)
	> = 20	180 (49%)
Location	Left-sided colon	154 (40%)
	Right-sided colon	229 (60%)

### Construction and evaluation of a prognostic signature based on APA

**Figure 2:**
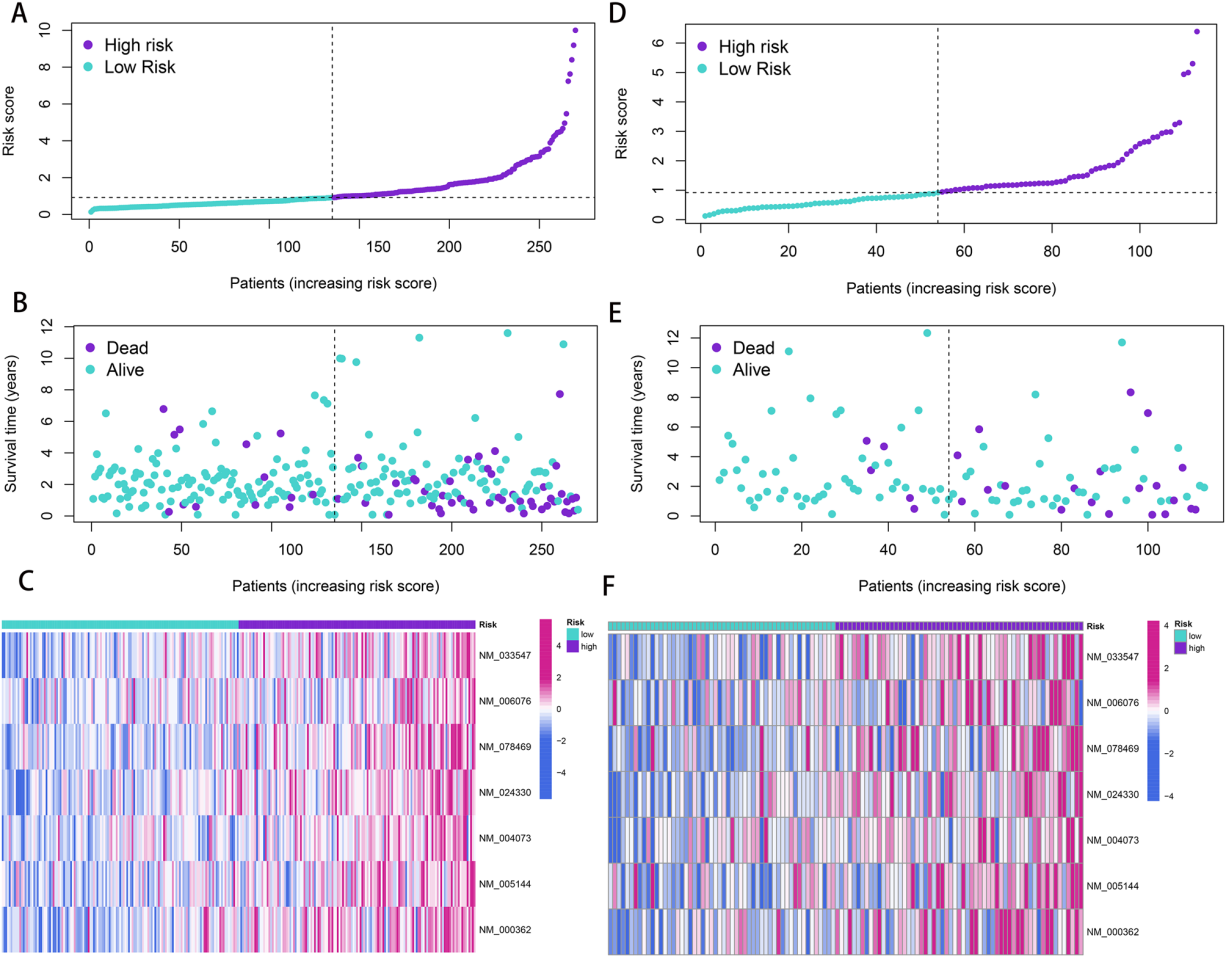
Development and validation of a four-APA-based prognostic signature. The risk curves, survival state diagrams, and risk thermographies in the training (**A**–**C**) and test (**D**–**F**) sets based on the signature.

**Figure 3:**
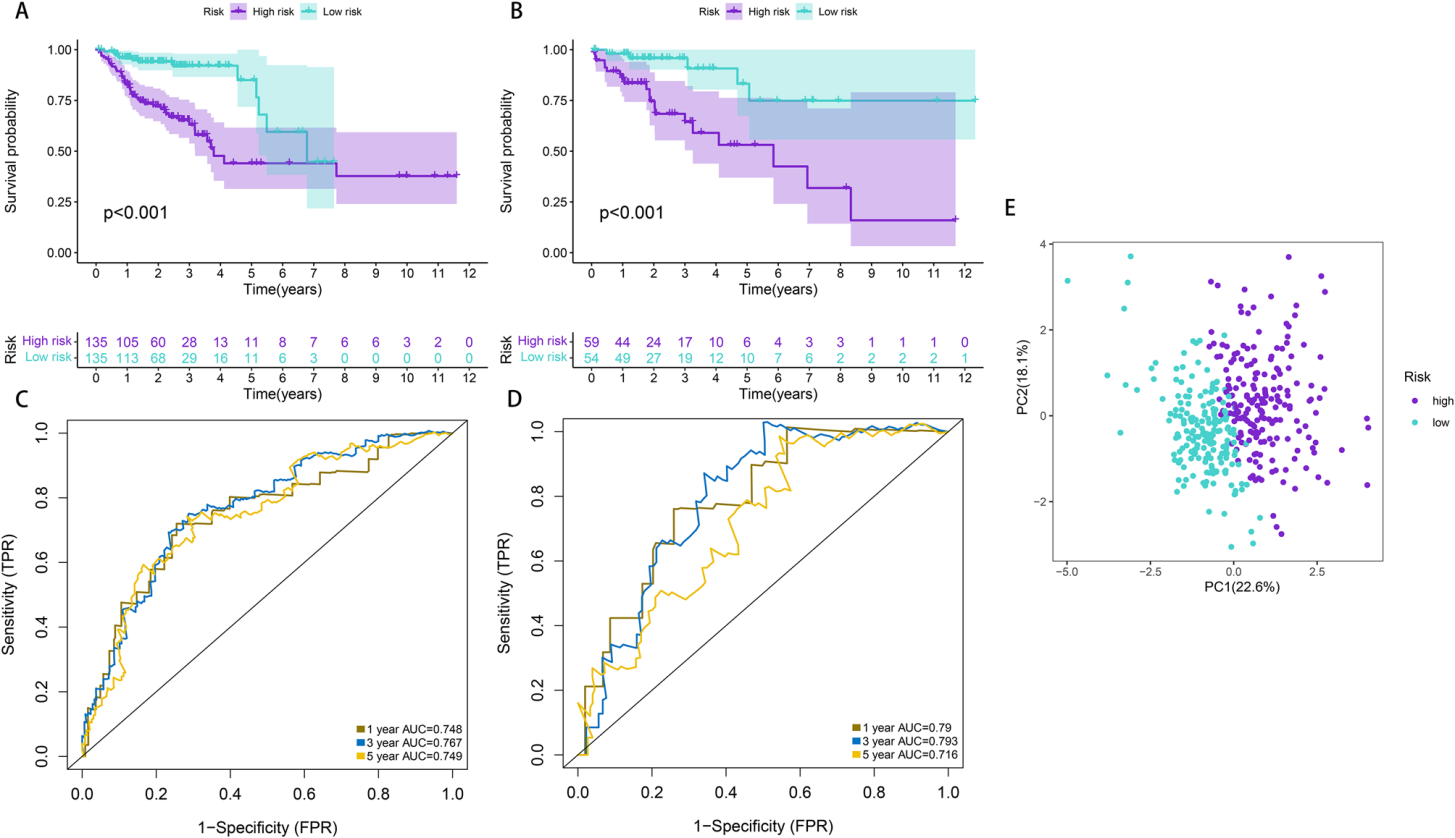
Prediction performances of the signature for CRC patients. (**A**,**B**) Survival curves in the training and test sets. (**C**,**D**) Time-dependent ROC curves for 1-, 3-, and 5-year OS predictions by the signature in the training and test sets. (**E**) PCA plot for CRC patients based on the risk groups.

**Figure 4:**
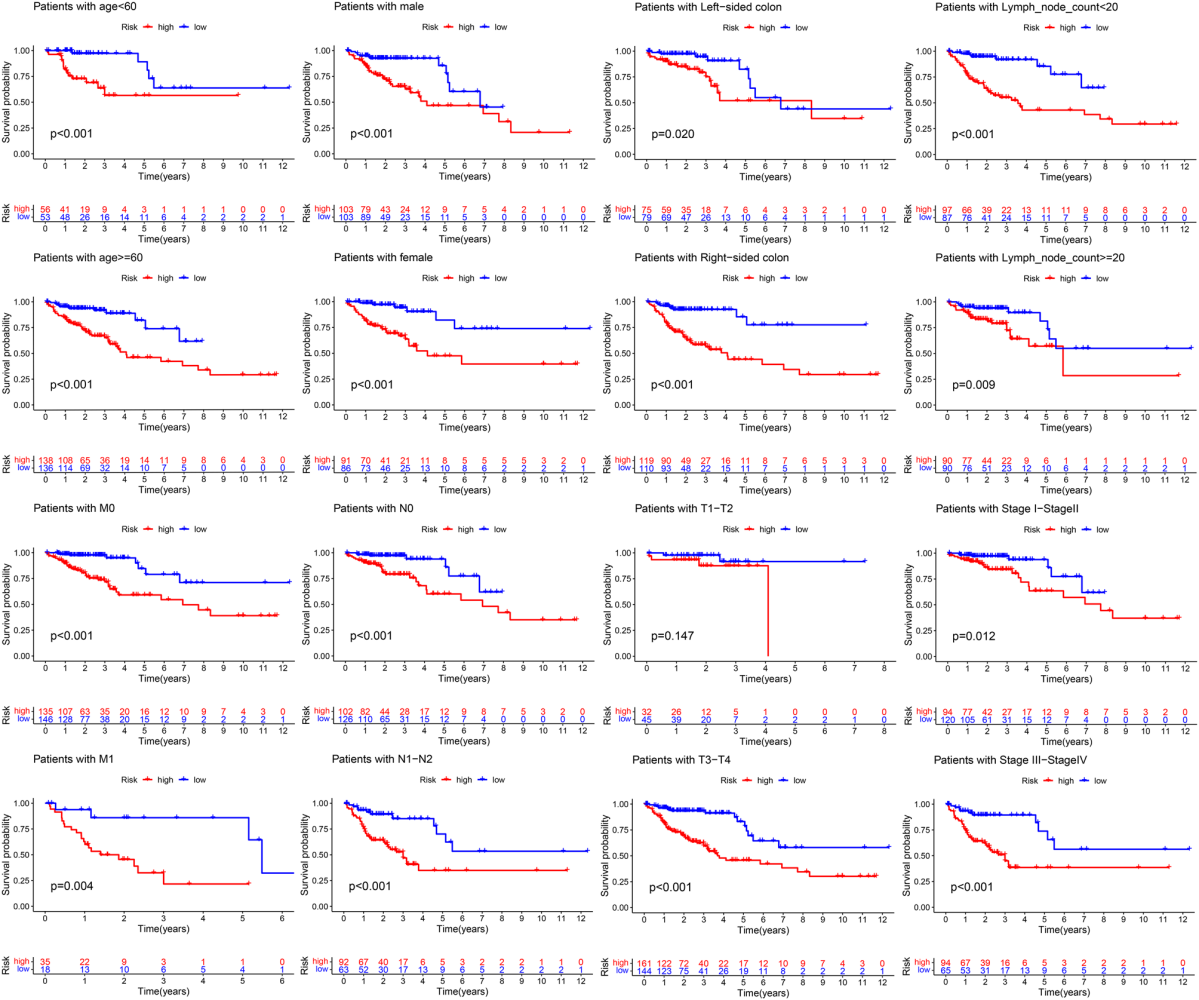
Survival curves in different clinical subgroups.

Based on the univariate Cox analysis, the top 20 most important APA events were screened further with the help of LASSO for the avoidance of overfitting of the signature (Fig. S2). Seven events were filtered out for the construction of the prognostic signature using multivariate Cox regression analysis from 18 APA events (Fig. 2A–C, Supplementary Table 5), including INTS4, AGFG2, BCCIP, SLC27A3, PLK3, HR, TIMP3. The following formula was utilized for calculating the risk scores: Risk score = (1.90654322* PDUI value of INTS4) + (3.58823772* PDUI value of AGFG2) + (1.497906193* PDUI value of BCCIP) + (2.491694604* PDUI value of SLC27A3) + (1.852487426* PDUI value of PLK3) + (2.567871349* PDUI value of HR) + (2.017448257* PDUI value of TIMP3). Patients were further sorted into two groups known as a high-risk group and a low-risk group based on their median risk scores. The K–M curves showed a significantly prolonged time to live (TTL) for patients in the low-risk group (Fig. 3A). The time-dependent ROC curve demonstrated the good predictive power of the signature over a 5-years span (Fig. 3C). (1-year AUC: 0.748, 3-year AUC: 0.767, 5-year AUC: 0.749). It is important to highlight that similar outcomes were obtained for the same models created in the test set (Figs. 2D–F; 3B,D; Supplementary Table 5). According to the PCA analysis, patients in the two risk groups were distributed in two separate directions (Fig. 3E, Supplementary Table 6). Furthermore, a clinical stratification analysis of prognostic prediction ability in the whole set was performed and adjusted by clinical features such as gender, age, and stage of the tumor. The findings demonstrated that in all clinical stratification subgroups, except for the TI-T2 subgroup, patients in the low-risk group had a better prognosis and survival status in comparison to the patients in the high-risk group (Fig. 4). These outcomes validated the accuracy and efficacy of the signature in predicting the prognosis of patients with CRC.

### Somatic mutation and copy number variants (CNVs) analysis

**Figure 5:**
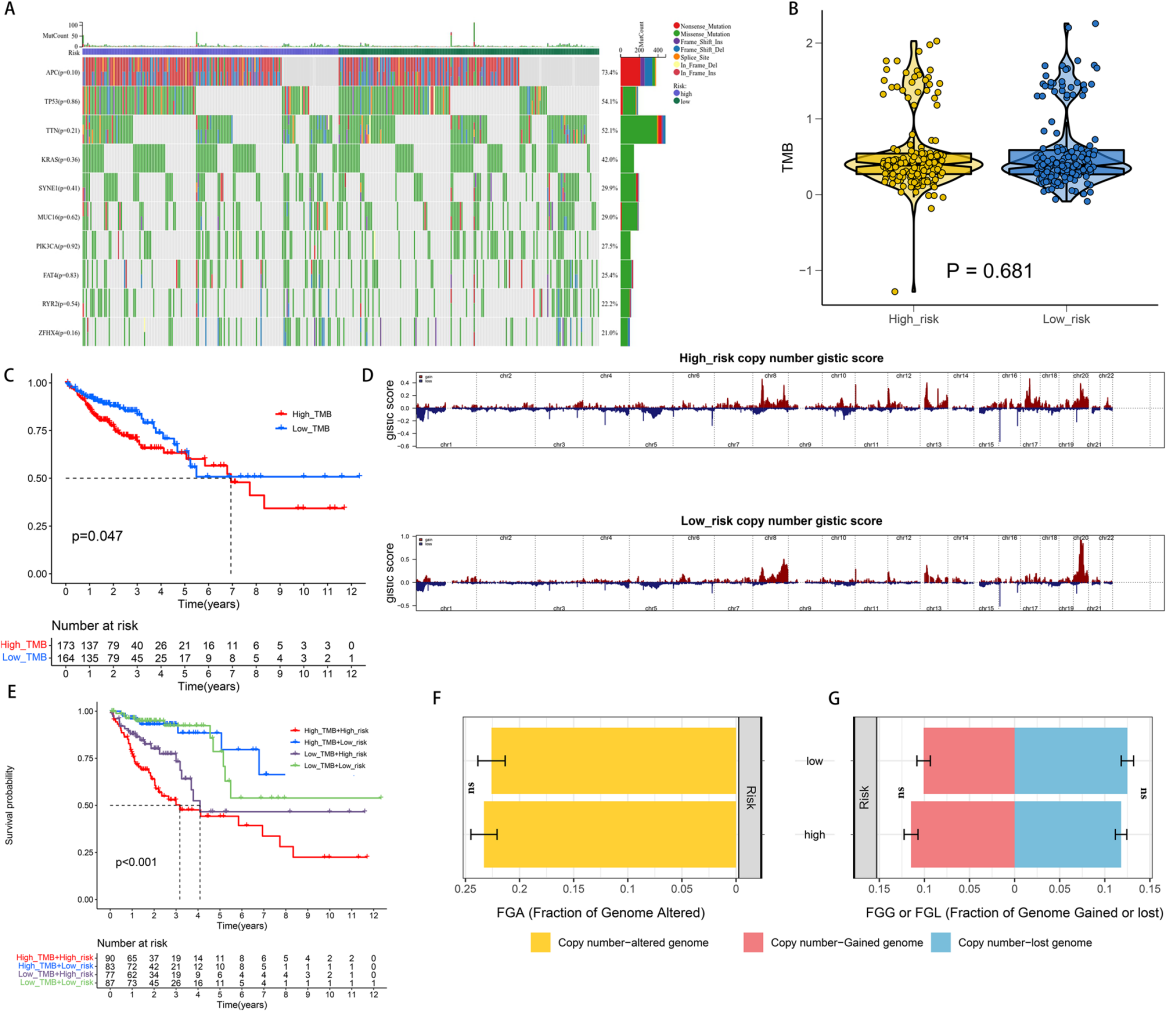
Integrated comparisons of somatic mutation and CNVs between high-risk and low-risk groups in the whole set. (**A**,**B**) Waterfall plots showing the mutation information of top 10 genes with the highest mutation frequency in high-risk and low-risk groups. (**B**) Distribution of TMB in two groups. (**C**) Survival curves for the high‐and low‐TMB groups. (**D**) Gene fragments profiles with amplification (red) and deletion (blue) among the two groups. (**E**) Survival curves for patients stratified by both TMB and signature. (**F**,**G**) Comparison of the fraction of the genome altered, lost, and gained between the two groups.

The distribution of mutations was identified and visualized in the high and low-risk groups (Fig. 5A). An integrated landscape of somatic mutations demonstrated the mutation patterns in the top 10 most frequently varying driver genes. However, there was no difference in the mutation number of these 10 genes between high-risk and low-risk groups. The relationship between the TMB and the constructed signature was also identified. Nevertheless, no obvious differences in the TMB levels between the two patient groups were noted (Fig. 5B, Supplementary Table 7). Patients were then divided into various subtypes based on their TMB scores as described before^29^. Survival curves presented that the higher the TMB scores, the shorter the OS time (Fig. 5C). For further identification of the validity of the risk scores and TMB, the synergistic effect of the two indicators in identifying the prognosis of patients with CRC patients was evaluated. As highlighted in the stratified survival curves, the low and high-risk groups demonstrated substantial prognostic-related differences in both low and high-TMB status subtypes (Fig. 5E). Furthermore, “GISTIC 2.0” was applied for decoding the amplification and deletion of CNVs on chromosomes. The outcomes indicated that the distribution of CNVs differed among the high and low-risk groups (Fig. 5D). Nevertheless, the genomic frequencies of CNVs did not differ greatly among the two groups (Fig. 5F,G, Supplementary Table 8).

### The evaluation of tumor immune microenvironment (TIM)

**Figure 6:**
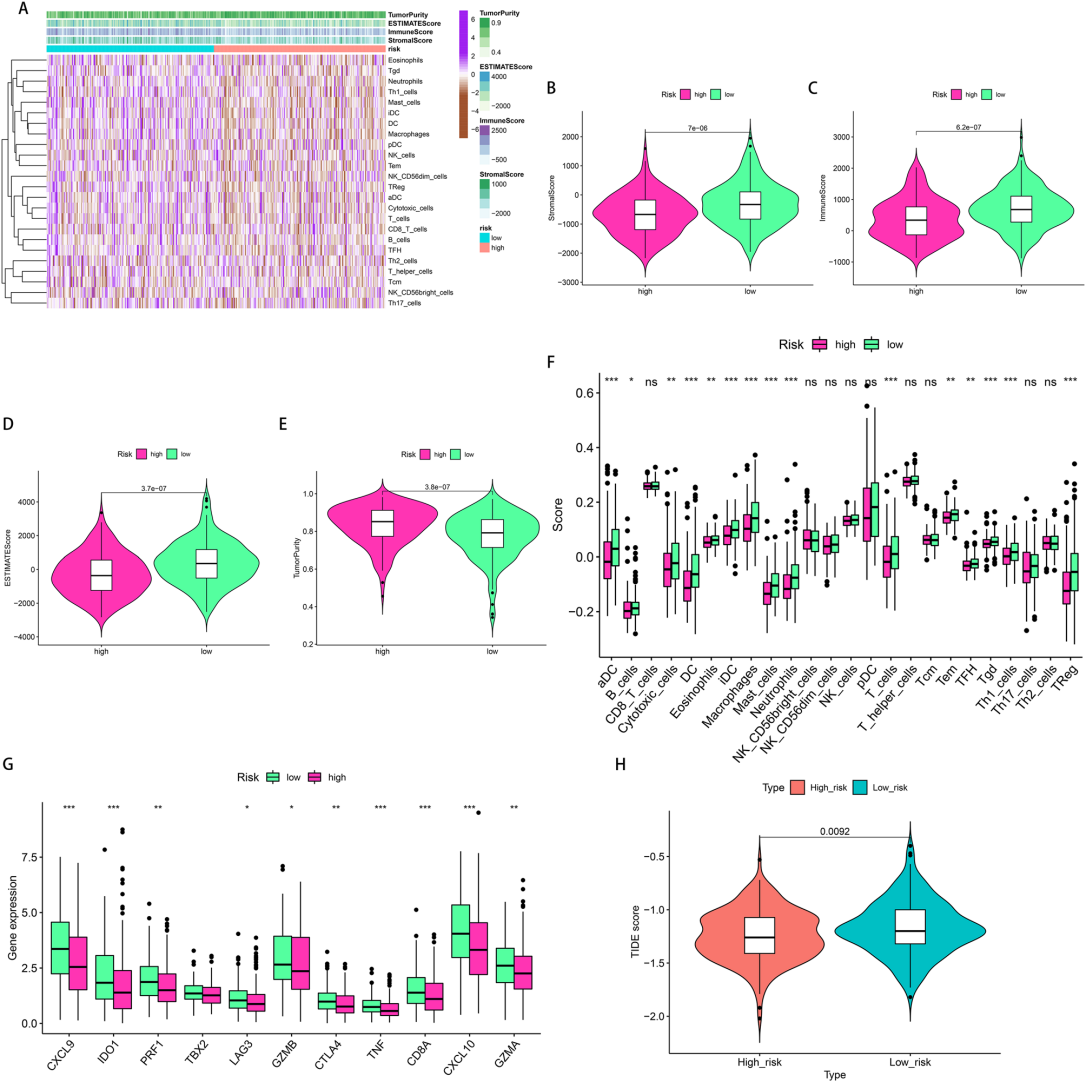
Estimation of the immune status and response to immunotherapy based on the signature in the high-risk and low-risk groups for the whole set. (**A**) Heatmap of the immune scores, stromal scores, tumor purity, ESTIMATE scores and immune-infiltrating cells in the two groups. (**B**–**E**) Violin plots for the immune scores, stromal scores, ESTIMATE scores, and tumor purity. (**F**,**G**) Boxplots of immune cells and immune checkpoints expression. (**H**) TIDE prediction difference in the two groups. *P < 0.05; **P < 0.01; ***P < 0.001; ns: no significance.

The TIM of all CRC samples was identified initially by measuring the immune scores, stromal scores, and ESTIMATE scores based on the ESTIMATE algorithm. According to the results, the immune scores, stromal scores, and ESTIMATE scores in the low-risk group were greatly increased as compared to those of the high-risk group (Fig. 6B–D, Supplementary Table 9). On the other hand, the tumor purity scores were elevated in the high-risk group (Fig. 6E, Supplementary Table 9). The heat map highlighted the difference in stromal/immune cell infiltration between both groups (Fig. 6A, Supplementary Table 9). For further analysis of the immune activity and tolerance of each group, differences in the immune cells and checkpoints were observed in the high and low-risk groups. A total of 15 kinds of immune cells, comprising aDC, Mast cells, T cells, etc., were greatly infiltrated in the low-risk group as compared to those in the high-risk group (Fig. 6F, Supplementary Table 10). Moreover, it was explored that the expression of most immune checkpoint-related genes was substantially elevated in the low-risk group, with an exception of the TBX2 gene (Fig. 6G).

### Efficacy prediction of immunotherapy and chemotherapy

The results stated above indicated the correlation between the signature with the immune status of patients. Consequently, it was hypothesized that the signature can potentially act as a marker for immunotherapy. Afterward, the TIDE algorithm validated the predictive efficacy of the signature for immunotherapy. Fascinatingly, it was discovered that immunotherapy was more effective in the high-risk group in comparison with the low-risk group (Fig. 6H, Supplementary Table 11). Additionally, the potential application of the risk signature in clinical chemotherapy was assessed by analyzing the differences in sensitivity of colon adenocarcinoma (COAD) to chemotherapeutic agents between the high and low-risk groups in the present clinical trial phase. Twenty-six compounds were found to have a greater effect on patients in the low-risk group (Fig. S3, Supplementary Table 12). These findings indicated that the signature has the potential to act as a predictor of sensitivity to radiotherapy and chemotherapy.

### Construction of a nomogram for independent prediction of prognosis

**Figure 7:**
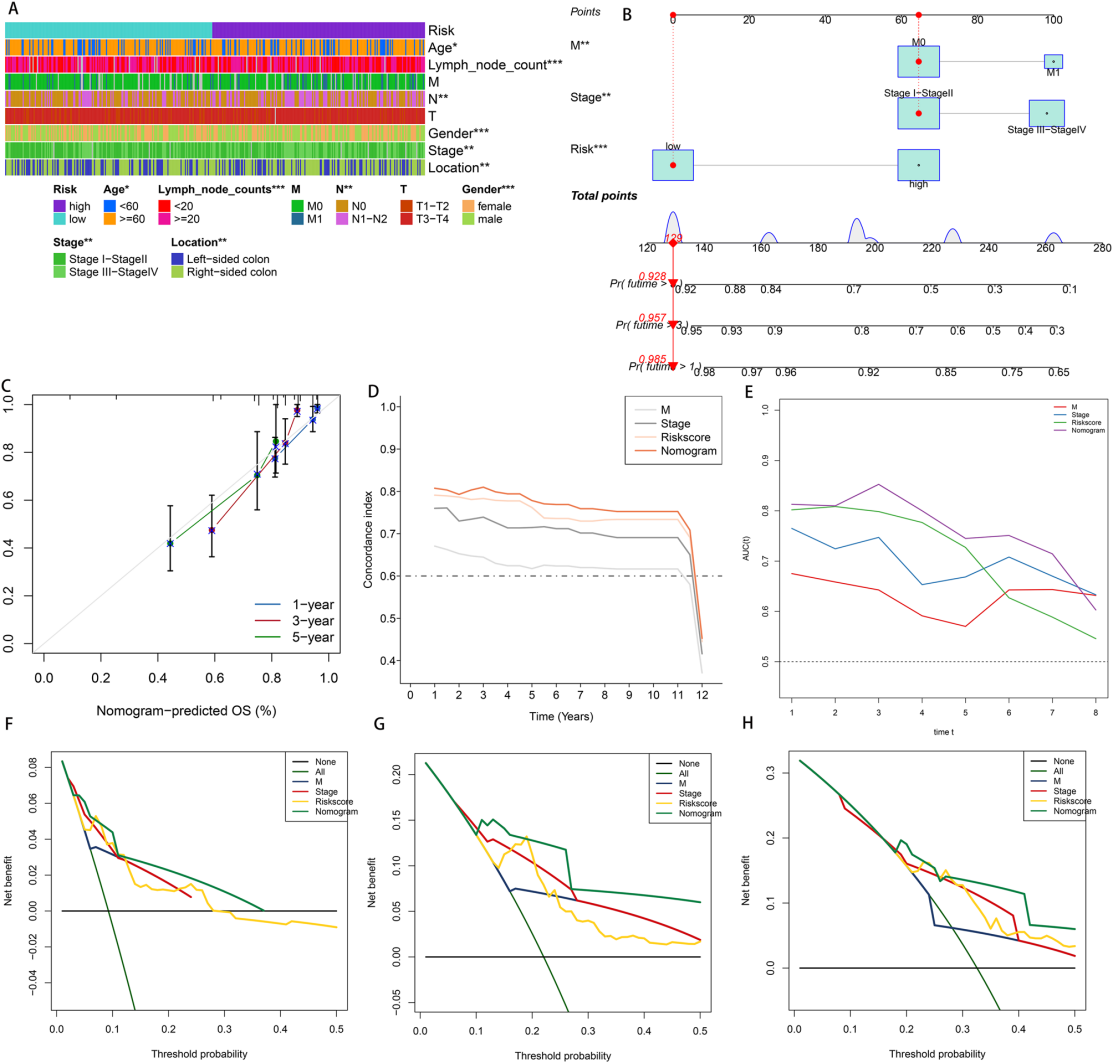
Correlation of signature with clinical factors and identification of the composite prognostic nomogram in the whole set. (**A**) Heatmap presents the distribution of clinical features and corresponding risk score. (**B**) Nomogram prediction of 1-, 3-, 5-year OS. (**C**) Calibration curves of observed and predicted probabilities for the nomogram. (**D**) Concordance index plot for the nomogram. (**E**) Time-dependent ROC curves for the nomogram. (**F**–**H**) DCA curves for the nomogram in 1-, 3-, 5-year OS. *P < 0.05; **P < 0.01; ***P < 0.001.

The clinical variables and the distribution of corresponding risk groups have been illustrated in Fig. 7A. The univariate and multivariate Cox regression analyses of risk scores and other clinical characteristics were performed for the assessment of their prognostic value for CRC, indicating that M, stage, and risk were independent prognostic factors for the prediction of the survival rate of patients with CRC (Table 2). To deliver a clinically working procedure for OS prediction in patients with CRC, with the help of independent risk factors, a nomogram was constructed (Fig. 7B). According to this consideration, individual patients were given a score for each prognostic factor, and adding up the higher total scores made the prognosis worse. Calibration curves, Time-AUC curves, and time-C-index all indicated the best performance of the nomogram as compared to other independent prognostic factors (Fig. 7C–E). Likewise, the DCA curve revealed that the nomogram also yielded the greatest net benefit (Fig. 7F–H).

**Table 2:** Univariate and multivariate Cox analysis of the clinicopathological features and signature with OS. Significant values are in bold.

Characteristics	Univariate Cox		Multivariate Cox	
	HR(95%CI)	*P* value	HR(95%CI)	*P* value
Age	1.141 (0.805–1.619)	0.458		
Lymph node count	0.768 (0.56–1.054)	0.103		
M	2.733 (1.922–3.888)	**< 0.001**	1.714 (1.122–2.62)	**0.013**
N	1.981 (1.452–2.272)	**< 0.001**	0.646 (0.302–1.384)	0.261
T	2.022 (1.121–3.648)	**0.019**	2.205 (0.789–6.161)	0.132
Stage	2.162 (1.557–3.001)	**< 0.001**	2.524 (1.052–6.054)	**0.038**
Gender	1.123 (0.829–1.522)	0.453		
Location	1.366 (0.989–1.888)	0.058		
StromalScore	0.863 (0.636–1.171)	0.344		
ImmuneScore	0.958 (0.706–1.3)	0.781		
ESTIMATEScore	0.891 (0.659–1.206)	0.456		
TumorPurity	1.106 (0.817–1.497)	0.516		
Risk	0.378 (0.261–0.546)	**< 0.001**	0.337 (0.216–0.525)	**< 0.001**

### Exploration and validation for APAs in signature

To explore the 3′UTR difference of APA in signature, we evaluated APAs by employing DaPars algorithm for each sample in open datasets GSE50760. Among these APAs, 5 APAs (INTS4, SLC27A3, BCCIP, PLK3, HR) were differentially expressed in CRC tissues, when compared with para-cancer tissues (Supplementary Table 14). Besides, TIM and therapies were validated in these patients with the same methods described above. The heatmap demonstrated the differential stromal/immune cell infiltration between the high and low-risk groups (Fig. S4A). As a result, the stromal and ESTIMATE scores of the low-risk group were markedly higher than those in the high-risk group (Fig. S4B,C). Conversely, the high-risk group scored higher in tumor purity (Fig. S4D). As for immune cell infiltration and immune checkpoint expressions, only aDC, neutrophils, Th1 cells, CXCL10, CXCL9, IDO1 and TBX2 were exceeded in the low-risk group (Fig. S4F,G). Finally, we further explored the relationship between risk scores and chemotherapy for this cohort. As shown in Fig. S5, low-risk patients appeared to be more susceptible to 16 drugs than high-risk patients. However, TIDE analysis indicated no differences in sensitivity to immunotherapy between the high and low-risk groups (Fig. S4H).

## Discussion

The occurrence and progression of CRC is a complicated procedure comprising various steps and genes. A study report has revealed that CRC cells have a particular biological behavior, such as an increased proliferative capacity, the recurrence tendency and the ability to metastasize^30^. Even though surgery, radiotherapy, and chemotherapy have contributed to the progress in the treatment of CRC, an effective procedure for the prediction of tumor progression or the prognosis of patients is not available at present^31,32^. The tumor-lymph node-metastasis staging (TNM staging) is a commonly used staging system, but it does not function in the accurate prediction of the prognosis of patients with CRC, and the prognosis pattern can be distinct even for CRC patients having the same stage of tumor^33^. Detailed research on the mechanisms involved in tumors has led to increasing benefits of prognosis prediction at the molecular level. For instance, Li et al. assessed the prognostic value of methylation regions in CRC and detected four differentially methylated regions, namely MUC12, TBX20, CHN2, and B3GNT7, as potential prognostic indicators for CRC and created a prognostic prediction score on their basis^34^. Moreover, many other reports have focused on transcriptional-level analysis, including research on mRNAs, long non-coding RNAs (lncRNAs), or miRNAs^35–37^.

APA is a major post-transcriptional regulatory mechanism that regulates the nuclear export, stability, and translation efficiency of mature mRNA^38,39^. New data indicated that aberrant APA patterns happening in different cancer types are involved in multiple oncogenic procedures in cancer occurrence and progression^9^. Earlier reports have indicated that CFIm25 promotes the protein expression of oncogenes, including IGF1R, by regulating their APA, thereby enhancing the proliferation and inhibiting the apoptosis of lung cancer cells^40^. The 3′UTR in the mRNAs of the PRELID1 gene was greatly shortened in ER-positive breast cancer tissues, which has the potential to improve the mRNA stability and translation efficiency of the PRELID1 gene substantially. Increased levels of PRELID1 expression led to enhanced tumor cell growth and substantially lowered the survival of cancer patients^41^. Currently there are several studies that have reported on global APA events in single cancer and pan-cancer. Analysis on raw transcriptome sequencing data of 17 cancer identified 1971 clinically relevant APA events, and 37 events may serve as common markers for multiple tumors^42^. For example, NDE1 was related to the clinical features (Subtype, Tumor/Normal, Stage and Survival) of 8 types of cancer. Besides, this study also suggested that APA profiles of some clinically actionable genes varied greatly among different tumor types, such as CTNNB1 and PIK3R1. This indicates the commonality and variability of some APAs in different tumors. Some studies have revealed the prognostic power of APA events in different cancers. Shortened 3′UTR of SMC1A was seen in lung adenocarcinoma and had a significant association with clinical prognosis^43^. Hu et al. have constructed two APA signatures for predicting OS and progress free-survival (PFS) of patients with sarcoma by the similar approaches to this study^44^. Notably, another study from our group that used a similar approach to build APAs signatures to predict OS and recurrence-free survival in breast cancer patients, suggesting more general prognostic roles for APAs^45^. For CRC, global APA patterns during cancer progression were detected and validated using 3′Seq and 3′polymerase chain reaction (PCR)^46^. However, this study about APA in CRC included only a small sample size and did not involve an analysis of the prognostic value of APA for patients. Considering the value of APA events in tumorigenesis and progression, they were investigated to gain comprehensive knowledge related to the prognostic value of APA events in CRC using TCGA analysis.

The CRC samples were systematically analyzed and a total of 197 survival-related APA events in 194 genes were obtained. GO and KEGG analyses of the parental genes for these events indicated that the enrichment events occurred frequently in cell adhesion molecule binding, perinuclear region of cytoplasm, Golgi vesicle transport, Pentose phosphate pathway, and other functions and pathways. These biological activities are also closely correlated with the invasion and metastasis of CRC cells and are regarded as the major damaging factors affecting the survival of patients with CRC. Furthermore, a correlation network was constructed between prognostic-related APA events and CRs to further probe into the regulatory role of CRs in APA events in patients with CRC. To explore the prognostic value of APA events, a prediction signature was developed in this study according to the prognosis-related APA events in the training set. Afterward, patients with CRC were sorted into high and low-risk groups based on their median risk scores, and a major difference in OS was revealed between both groups. It was observed that the 1, 3, and 5-year AUC values for patients with CRC were all higher than 0.75, showing good predictive ability. The signature showed similar predictive potential in the test set, highlighting its good utility and reproducibility in predicting OS in patients with CRC. However, for the RNA expression level of the parental genes in the signature, only AGFG2 was associated with patient prognosis, which illustrated the independence of APAs as indicators (Supplementary Table 13).

Our results revealed that the difference existed in 3′UTRs of 5 APAs between tumor and normal samples. This suggests the APAs in the signature are more likely to have potential biological value. The majority of the PDUI value of 5 APAs was lesser in tumor samples, which supported previous findings that shortening of the 3′UTR is more likely to occur in tumors (9). Unfortunately, studies on the biological functions of APAs events in the current signature are blank. As mentioned above, Changes in APA may affect gene transcription, translation and stability, thus affecting the biological functions of genes. Therefore, we can indirectly reflect the potential biological functions of APAs from the roles of parental genes of APA in tumors. A variety of genes among the APA parental genes in the signature were observed to play a significant role in the biological function of CRC progression. BCCIP, an interacting protein of BRCA2 and CDKN1A^47^, is strongly expressed in the progression of CRC^48^. Xu et al. revealed that Celecoxib affected the function of p53 and inhibited recovery from the damage caused by irradiation by upregulating the expression of BCCIP. Moreover, Celecoxib elevated the radiosensitivity of CRC cells by regulating the expression of genes, including p21 and Cyclin B1, in a COX-2 independent manner^49^. PLK3 belongs to the serine-threonine kinase family and has a significant role in the cell cycle^50^. In case of DNA damage, PLK3 has been observed to phosphorylate and interact with WT in S20 and P53, which activates tumor suppressors and induces apoptosis^51^. PLK3 inhibits cancer cell growth and suppresses cellular glucose metabolism through the heat shock protein 90 (HSP90)/signal transducer and activator of transcription 3 (STAT3)/hexokinase 2 (HK2) pathways^52^. Consequently, low PLK3 expression in CRC tissues is correlated with a poor prognosis. Tissue inhibitor of metalloproteinases 3 (TIMP3), a member of the TIMP family, has been noted to inhibit tumor growth, angiogenesis, invasion, and metastasis^53^. TIMP3 was observed to enhance apoptosis susceptibility and facilitate apoptosis by stabilizing tumor necrosis factor-alpha (TNF- α) receptors on the surface of CRC cells^54^. Additionally, the overexpression of TIMP3 may reduce vascular density, promote apoptosis and inhibit malignant behaviors, including migration, invasion, and tumor growth of CRC cells^55^. Collectively, these APA events are closely correlated with CRC metastasis and the survival of patients and can act as potential prognostic and therapeutic targets for CRC.

Various clinical information has shown a correlation between genetic alterations and the responsiveness to immunotherapy^56,57^. However, the number of mutations of the top 10 most frequently varying driver genes was not statistically different between the high and low risk groups in the current study. For the TMB, it did not differentiate between patients' risk subtypes but did discriminate between the prognosis of patients. Subsequent stratified survival curves indicated the prognostic predictive ability of the risk scores independent of TMB, indicating that TMB and risk scores signify various aspects of immunobiology. Additionally, the risk scores and CNVs highlighted a different distribution between high and low-risk groups, but no substantial differences were found in the genome where CNVs occurred.

The tumor immune microenvironment (TIM) is a significant factor in the progression of tumors, in which the immune system protects tumor cells from the immune barrier. Tumor immune editing and resistance results in immune escape, promoting tumor cell proliferation, invasion, and metastasis^58,59^. Hence, we hypothesized and studied the correlation between APA events and TIM, which could provide potential prognostic and therapeutic targets for CRC patients. It was observed that the high-risk group showed decreased immune and stromal cell scores and presented low immune cell infiltration and immune checkpoint levels, while the low-risk group had low tumor purity and indicated considerable immune cell infiltration and increased immune checkpoint levels. These findings indicated that the signature can reveal the immune status of patients with CRC, which are corresponding to the results of enrichment analysis (GO term T cell receptor binding and the KEGG Pathway Th1/Th2 cell differentiation). However, these results are only based on the estimation of cell characteristic gene expression, and the expressed genes may come from infiltrating cells rather than tumor epithelial cells. We will carry out more accurate detection of cell sorting or flow cytometry to verify our results, considering that the signature may function to distinguish the stromal and epithelial signal. Furthermore, the TIDE algorithm showed lower scores in the high-risk group, suggesting that patients in the high-risk group were more sensitive to immunotherapy. Moreover, 26 potential antitumor agents associated with the signature were identified in this study, which provided an additional reference for antitumor therapy for patients with distinct risk levels. Since the sample size is relatively small in GSE50760 cohort, the results about TIM and prediction of treatment have not been very consistent. Nevertheless, the overall trend of difference in the two groups is consistent. As our results were obtained from the bioinformatic analysis, additional clinical studies are needed for further confirmation.

At the end of this study, the nomogram was constructed for better prediction of the survival of patients with CRC and to visualize the predicted results, providing the additional direction for patient adherence and treatment outcomes. Moreover, the effectiveness and validity of the nomogram were compared with independent prognostic indicators, indicating that the nomogram can deliver better prognostic ability and more net gaining than other independent indicators in the clinical setting. The outcomes of this study indicated the reliable prognostic accuracy of the nomogram on the basis of seven APA events.

Certain limitations remained in the current study. First of all, the APA event set was generated using relatively lax criteria. These screening criteria allowed us to categorize a large number of potentially important APA events, but the reliability of this study could have been affected adversely. Moreover, due to the unavailability of other publicly present datasets, the signature used in this study was only verified internally. Evidently, independent datasets and prospective studies are required for further validation of the reliability of this signature. Lastly, the biological activity of these APA events in CRC needs to be examined in vivo and in vitro.

## Conclusion

Briefly, this study identified the substantial prognostic value of APA events in CRC and constructed a reliable signature for the prediction of the survival results of patients. Moreover, the signature allowed the differentiation of patients' immune status and stratification of immune/chemotherapy-sensitive patients with biological evidence. In general, this study enhanced our knowledge regarding the detailed molecular mechanisms of CRC and provided a list of potential biomarkers and therapeutic targets for CRC.

## Supplementary Information

Supplementary Figures.

Supplementary Tables.

## Data Availability

Raw data related to all AS events were provided by the UCSC (University of California-Santa Cruz) Xena database (https://xena.ucsc.edu). Then, the RNA sequencing (RNA-seq), somatic mutations, copy number variants (CNVs), and clinical information of the CRC cohort were obtained from The Cancer Genome Atlas (TCGA) database (https://portal.gdc.cancer.gov/).
