# Supplementary material for: Alternative polyadenylation associated with prognosis and therapy in colorectal cancer
Source: Sci Rep. 2022 Apr 29;12:7036. doi: 10.1038/s41598-022-11089-9 (PMC9054804; doi:10.1038/s41598-022-11089-9)
Supplement: Supplementary file 1 — Supplementary Figures. [file 41598_2022_11089_MOESM1_ESM.pdf]

Supplementary Figures

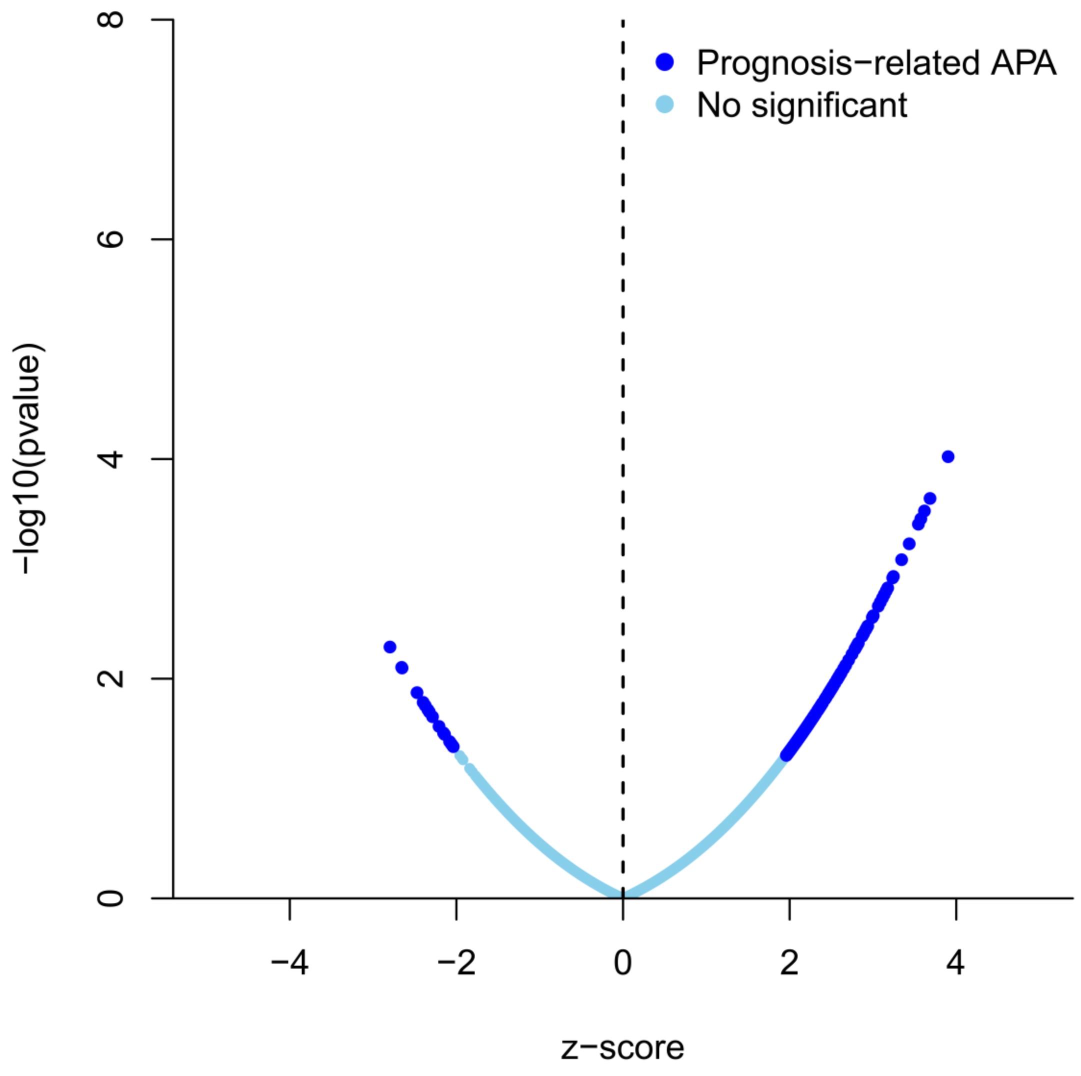

Figure S1. Volcano plot of prognosis-related APA events.

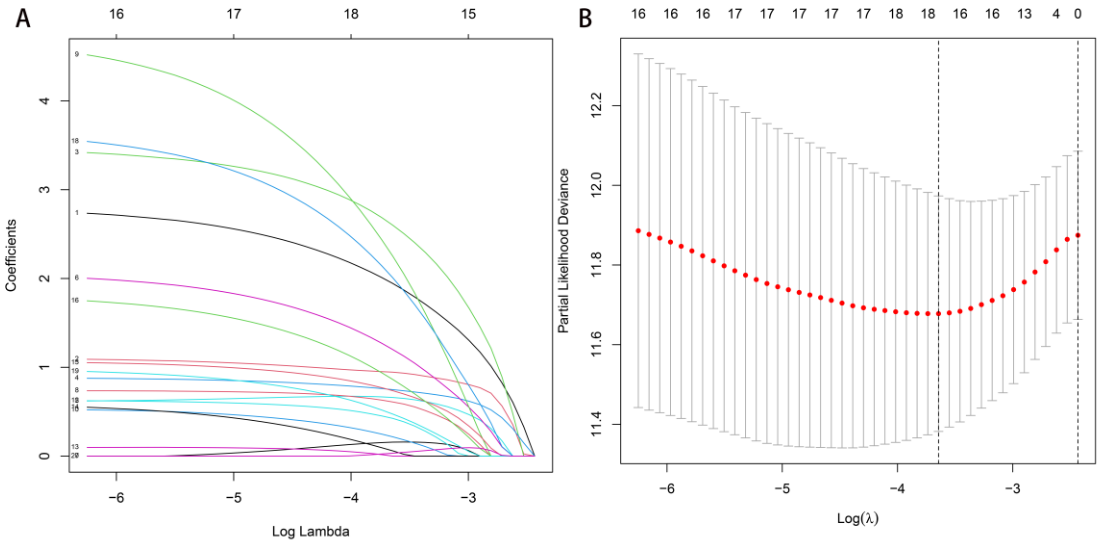

Figure S2. The key APA events were identified using LASSO regression. (**A)** Selection of the optimal parameter (lambda) via 5 times cross–validation. (**B)** LASSO coefficient profiles of the top 20 prognosis-related APA events.

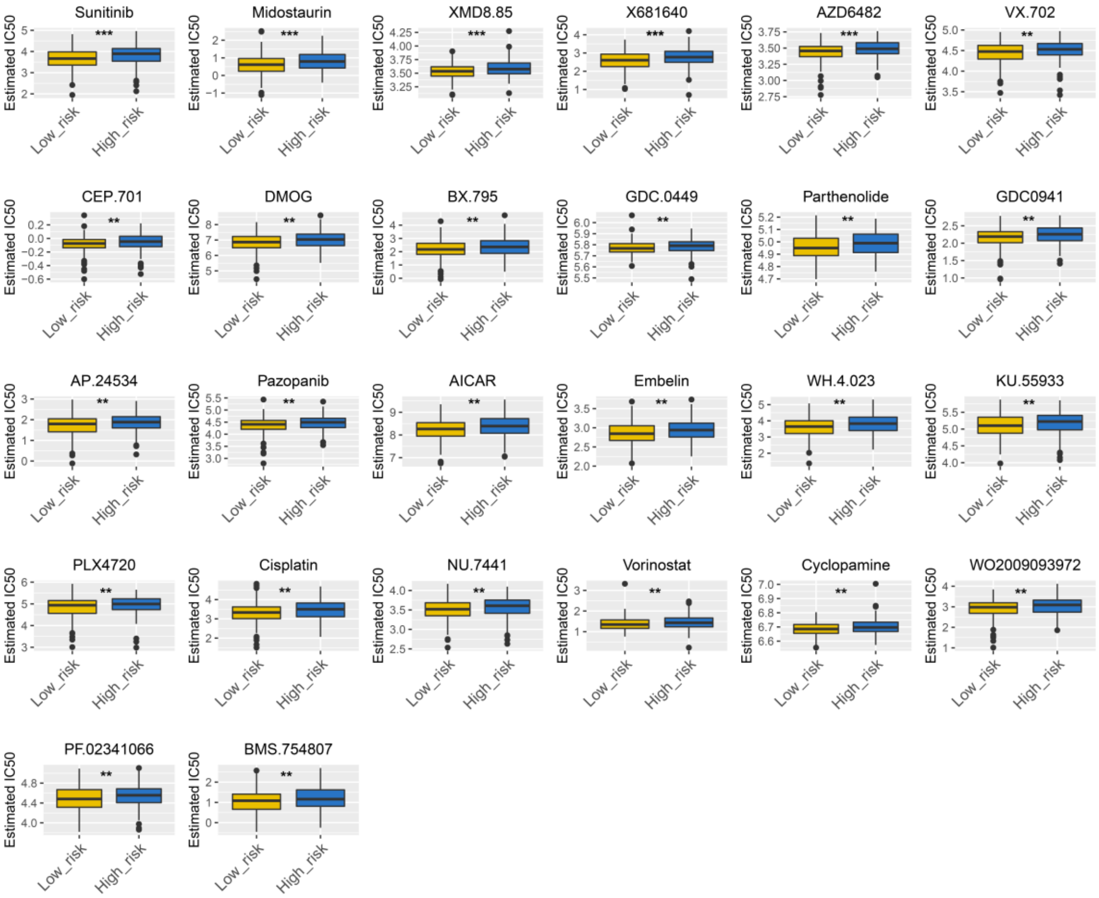

Figure S3. Boxplots of estimated IC50 values of potential compounds between high-risk and low-risk groups for TCGA cohort. *P <0.05; **P <0.01; ***P <0.001.

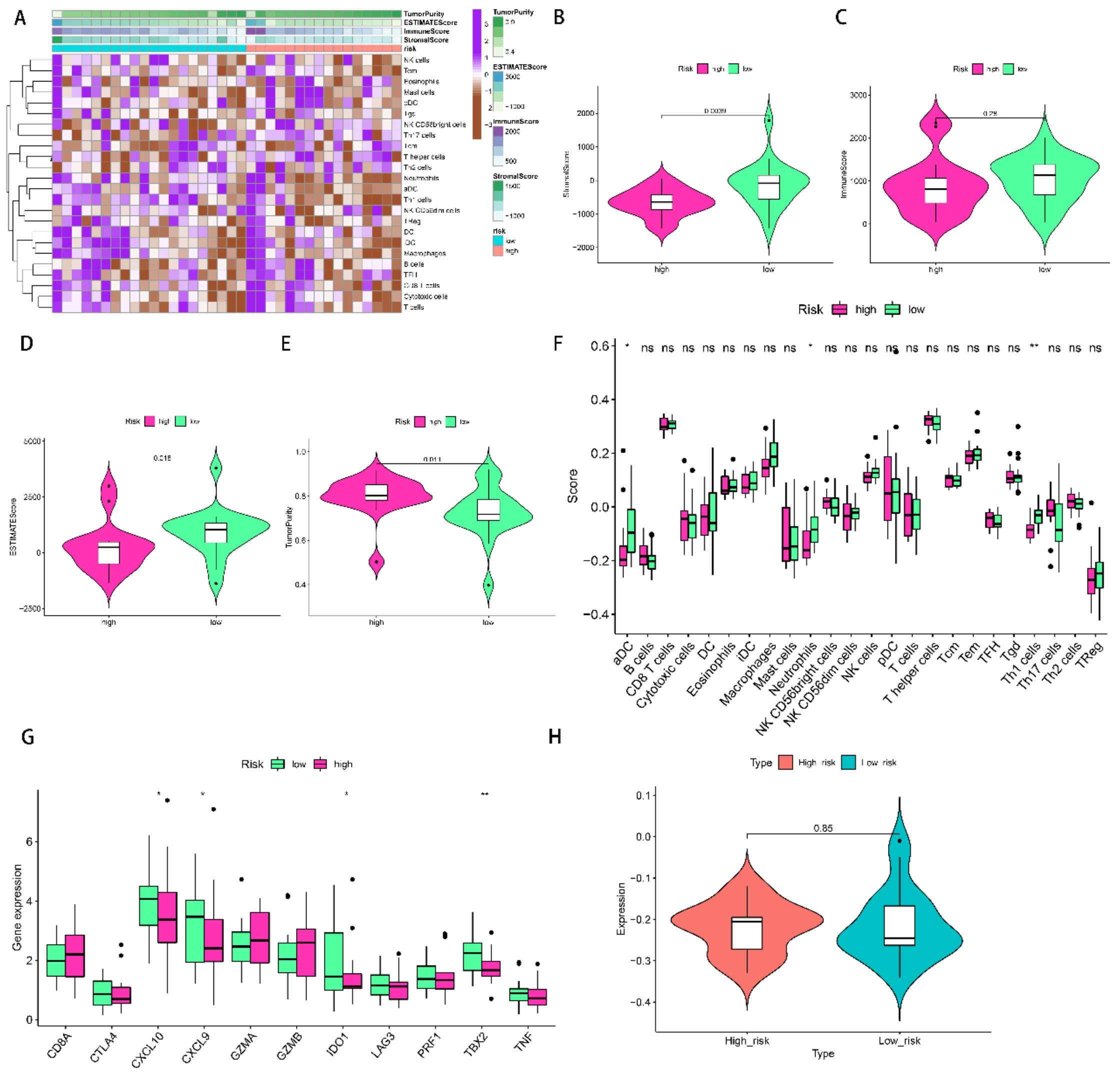

Figure S4. Estimation of the immune status and response to immunotherapy based on the signature in the high-risk and low-risk groups for the GSE50760. (A) Heatmap of the immune scores, stromal scores, tumor purity, ESTIMATE scores and immune-infiltrating cells in the two groups. (B-E) Violin plots for the immune scores, stromal scores, ESTIMATE scores, and tumor purity. (F-G) Boxplots of immune cells and immune checkpoints expression. (H) TIDE prediction difference in the two groups. *P <0.05; **P <0.01; ***P <0.001; ns: no significance.

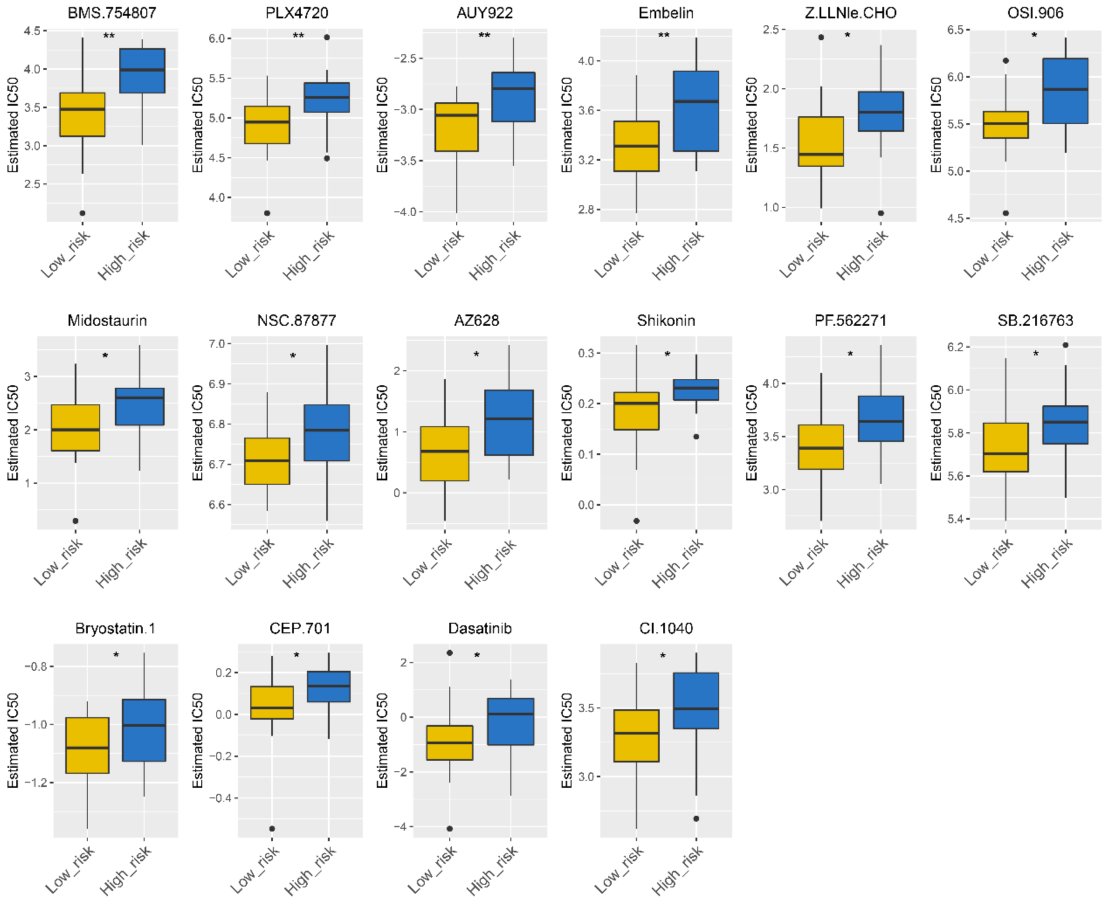

Figure S5. Boxplots of estimated IC50 values of potential compounds between high-risk and low-risk groups for GSE50760 cohort. *P <0.05; **P <0.01; ***P <0.001.
